# A look-up table based approach to characterize crystal twinning for synchrotron X-ray Laue microdiffraction scans

**DOI:** 10.1107/S1600576715004896

**Published:** 2015-04-25

**Authors:** Yao Li, Liang Wan, Kai Chen

**Affiliations:** a Center for Advancing Materials Performance from the Nanoscale (CAMP-Nano), State Key Laboratory for Mechanical Behavior of Materials, Xi’an Jiaotong University, Xi’an, Shaanxi 710049, People’s Republic of China

**Keywords:** twinning, synchrotron X-ray Laue microdiffraction, crystal orientation maps, look-up tables

## Abstract

An automated method has been developed to characterize the type and spatial distribution of twinning in crystal orientation maps from synchrotron X-ray Laue microdiffraction results. The method relies on a look-up table approach. Taking into account the twin axis and twin plane for plausible rotation and reflection twins, respectively, and the point group symmetry operations for a specific crystal, a look-up table listing crystal-specific rotation angle–axis pairs, which reveal the orientation relationship between the twin and the parent lattice, is generated. By comparing these theoretical twin–parent orientation relationships in the look-up table with the measured misorientations, twin boundaries are mapped automatically from Laue microdiffraction raster scans with thousands of data points. Taking advantage of the high orientation resolution of the Laue microdiffraction method, this automated approach is also applicable to differentiating twinning elements among multiple twinning modes in any crystal system.

## Abstract 2

An automated method has been developed to characterize the type and spatial distribution of twinning in crystal orientation maps from synchrotron X-ray Laue microdiffraction results.

## 1. Introduction   

Crystal twinning, referring to two or more homogeneous individuals oriented by a defined symmetry element outside the crystal’s point group, is commonly observed during crystal growth, recrystallization annealing, phase transformation (Li *et al.*, 2014 ▶) and plastic deformation (Cahn, 1954 ▶). According to the symmetry operation added into the crystal, twinning can be cataloged into three types, including rotation, reflection and inversion twins (Koch, 2004 ▶). The twin axis, twin plane and twin center are the most important elements for the three twinning types, respectively. Twinned crystals usually exhibit unique effects distinct from their untwined counterparts in the aspects of mechanical behavior (Christian & Mahajan, 1995 ▶; Salem *et al.*, 2005 ▶; Kaschner *et al.*, 2006 ▶), texture evolution (Brown *et al.*, 2005 ▶; Proust *et al.*, 2007 ▶), and other physical properties. Often, because they have a lower interfacial energy than ordinary grain boundaries (OGBs), twin boundaries (TBs) have a major impact on yield stress (Konopka & Wyrzykowski, 1997 ▶), work hardening (Bouaziz *et al.*, 2008 ▶) and fatigue cracking initiation (Heinz & Neumann, 1990 ▶), and cause migration of twin walls in some shape memory alloys induced by external magnetic fields (Likhachev & Ullakko, 2000 ▶). It is therefore essential to characterize the type, element and spatial distribution of twinning in a crystalline material to understand and predict its mechanical behavior.

As crystal twinning occurs commonly at the nanometre to micrometre scale, conventional neutron and X-ray diffraction cannot be employed to map the spatial distribution of crystal twinning owing to their limited spatial resolution. Although electron diffraction based on transmission electron microscopy (TEM) has an exceptional advantage of spatial resolution, its angular resolution is poor, and the viewing area is usually only a couple of micrometres. With the emergence of microdiffraction techniques such as electron backscatter diffraction (EBSD) and synchrotron polychromatic X-ray microdiffraction (µXRD), the microstructure (such as phase distribution, crystallographic orientation, local microstrain and defect distribution) of heterogeneous materials can be studied on a length scale spanning intra-granular and inter-granular dimensions. EBSD provides high spatial resolution (down to the nanoscale) but relatively low angular/orientation resolution (1–0.5° in the conventional setup, 0.1° in high-resolution mode) (Wilkinson *et al.*, 2006 ▶). Additionally, in order to capture high-quality diffraction patterns, it is crucial to make the EBSD sample surface free of contaminants, oxidation layers and charge accumulation. However, with the technique of µXRD, one can achieve an angular resolution down to 0.01° and a 10^−4^ strain resolution with proper calibration (Budai *et al.*, 2003 ▶; Chen *et al.*, 2010 ▶). Besides, it is much easier to prepare samples for µXRD than for EBSD because of the stronger penetration ability of X-rays compared to backscattered electrons.

However, analysis and interpretation of µXRD data are less advanced than those for TEM and EBSD. Though people have characterized the type and/or distribution of twinning by µXRD before (Savytskii *et al.*, 2003 ▶; Wang *et al.*, 2010 ▶; Guo *et al.*, 2011 ▶; Mun *et al.*, 2011 ▶; Li *et al.*, 2014 ▶), the need for an automatic approach that is valid for all seven crystal systems still exists (Wang *et al.*, 2013 ▶). In this study, the *X-ray Microdiffraction Analysis Software* (*XMAS*) (Tamura, 2014 ▶) is used to index Laue diffraction patterns and measure the crystallographic orientation. Then a ‘look-up’ approach is developed to determine and map the crystal twinning by comparing the orientation outputs of a µXRD raster scan with a calculated look-up table, which is established taking into account geometric relationships between twin pairs and the rotational symmetry of the point group.

## 2. Methodology   

It is noted that the tensors referred to in this article are denoted as three-dimensional square matrices. In the case of hexagonal and trigonal systems, three-digit Miller index notation is used throughout the mathematical calculations, and the final outcomes are entirely converted to four-digit Bravais–Miller indices.

According to Friedel’s law, conventional diffraction techniques without anomalous scattering (Rossmann, 1961 ▶) are unable to determine the existence of an inversion center in a crystal from the intensities of its diffraction pattern. This is because conventional diffraction methods impose an additional inversion center on the diffraction pattern. As a result, inversion twins cannot be identified with the conventional Laue diffraction method, and thus only rotation and reflection twins are discussed in this paper. Moreover, because of the addition of the inversion center, the method employed here is insensitive to the chirality of the crystals, and thus reflection twin cannot be detected in crystals with noncentrosymmetry. For example, the Brazil twin in quartz is formed by reflecting the parent crystal across the {11
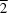
0} mirror plane. In this case neither the position nor the intensity of the Laue diffraction peaks will change with the formation of the twin domain, unless the anomalous scattering technique is applied (González-Mañas *et al.*, 1993 ▶). Therefore, reflection twins can only be identified in crystals with centrosymmetry. Their Laue groups are listed in Table 1 ▶.

It can be proven that, for centrosymmetric crystals, the relationship between a reflection twin and its parent can be expressed by a pure rotation. The rotation angle–axis pair associated with the reflection twin is defined by the twin plane, lattice parameters and crystal point group (for details see §2.3). A rotation twin by definition is also described through a rotation axis and angle. We therefore will describe both types of crystal twinning by look-up tables in terms of rotation angle–axis pairs in this paper. By comparing the experimental observed misorientation between each two adjacent positions in a µXRD scan with the calculated ones, twin boundaries can be detected within a suitable angular tolerance, and twin type and element can be uniquely determined.

### 2.1. Treatment of rotations   

**Figure 1:**
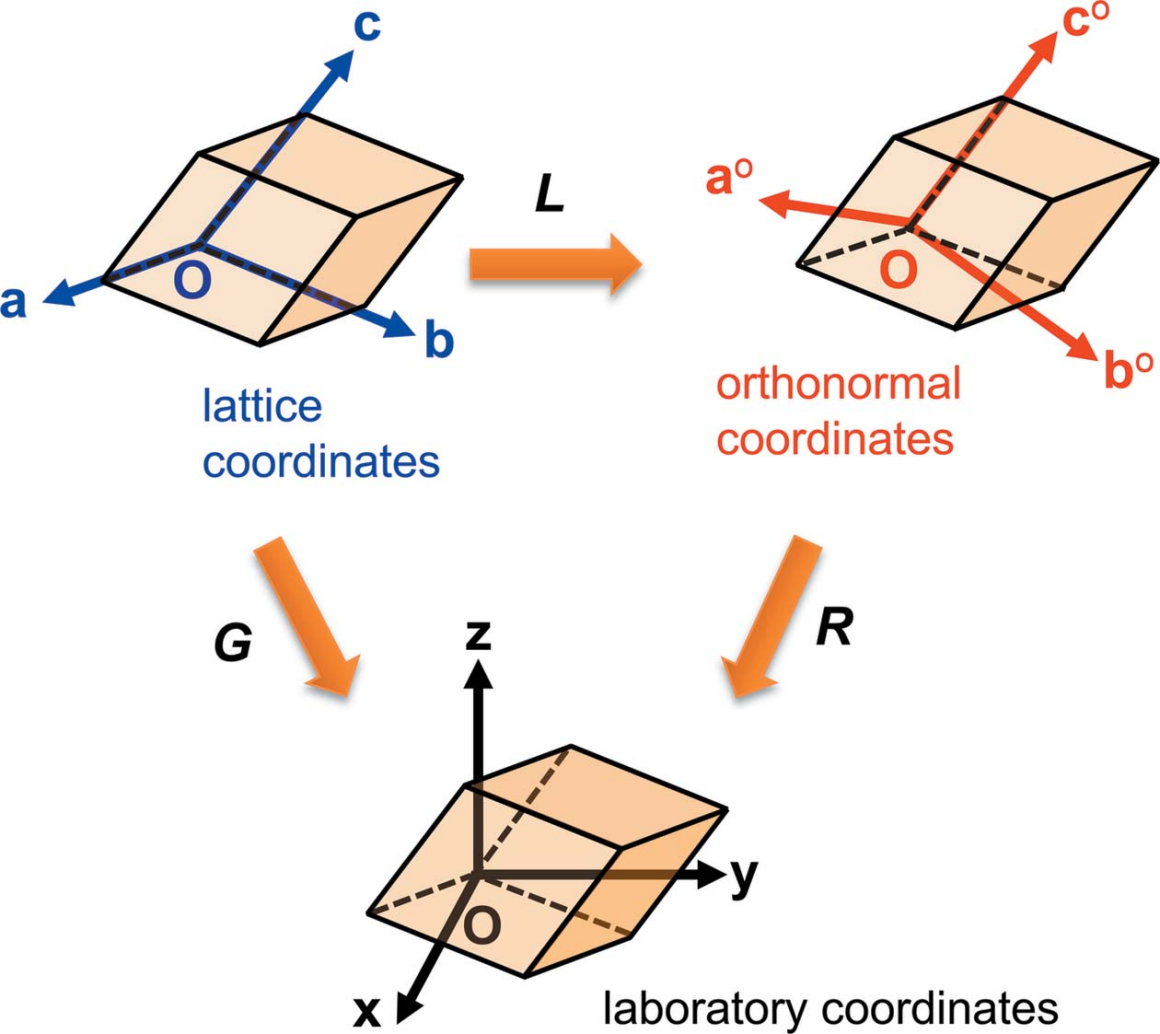
The definition and mutual relationship of the three coordinate systems referred to in this article.

The orientation of a crystal can be represented as a matrix, and in this article we follow the definition used by *XMAS* and call it orientation matrix ***G***:
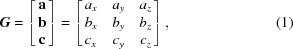
where row vectors **a**, **b** and **c** are the basis lattice vectors while **x**, **y** and **z** are the unit vectors of a Cartesian laboratory coordinate system (**O-xyz**). On the other hand, it is often convenient to define another Cartesian coordinate system (**O-a** ^o^ **b** ^o^ **c** ^o^, and we call it orthonormal coordinates in this article) which is associated with the crystal lattice coordinate system **O-abc**, so that the orientation for a crystal from any of the seven crystal systems can be defined as a pure rotation that brings **O-xyz** into **O-a** ^o^ **b** ^o^ **c** ^o^. Here we define that the two coordinates of **O-abc** and **O-a** ^o^ **b** ^o^ **c** ^o^ share the same origin and the **c** ^o^ axis is collinear with the **c** axis, **b** ^o^ is perpendicular to the *ac* plane, and **a** ^o^ lies hence in the *ac* plane to fulfill the right-handed criterion (Matthies *et al.*, 1988 ▶); **a** ^o^, **b** ^o^ and **c** ^o^ are unit vectors. Therefore, the transformation matrix ***L*** that brings **O-a** ^o^ **b** ^o^ **c** ^o^ to **O-abc** has the following form:
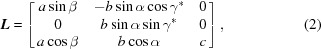
where *a*, *b*, *c* are the edge lengths of the unit cell, α, β, γ are the angles between the edges, and γ* is the angle between the reciprocal unit axes a* and b*. The relationships between **O-abc**, **O-a** ^o^ **b** ^o^ **c** ^o^ and **O-xyz** are displayed in Fig. 1 ▶. Consequently, the [*uvw*] direction in the lattice coordinate system can be transformed into the [*u* ^o^ *v* ^o^ *w* ^o^] direction referred to the orthonormal coordinate system as
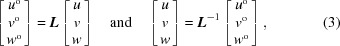
where the superscript −1 denotes the inverse matrix. Similarly, the (*h* ^o^ *k* ^o^ *l* ^o^) plane in the orthonormal coordinate system can be obtained from the (*hkl*) plane in the lattice coordinates by (He & Jonas, 2007 ▶)
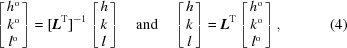
where the superscripts T and −1 denote the transpose and inverse of a matrix, respectively.

For a mathematical representation of rotations, one can alternatively choose rotation matrices, rotation angle–axis pairs, Euler angles or unit quaternions. Here we prefer to use unit quaternions for all calculations but angle–axis pairs to display the final results, because the former are more accurate in computing while the latter are more transparent in expressing the rotation. The unit quaternion **q** = [*q*_0_ *q*_1_ *q*_2_ *q*_3_] corresponding to an angle–axis pair {θ, [*u*_1_ *u*_2_ *u*_3_]} has the form of
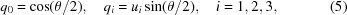
where *q*_0_ and *q_i_* are the scalar and vector parts, respectively. Accordingly, the rotation matrix ***R*** in terms of unit quaternion **q** is
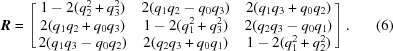
The mutual transformation between these rotation representations can be found elsewhere (Brannon, 2002 ▶).

### 2.2. Calculating look-up tables for rotation twins   

A rotation twin is defined as two twin components brought into coincidence by rotating the parent lattice by a well defined angle θ (360°/*n*, *n* = 2, 3, 4, 6) around a well defined axis **t** (twin axis) (Koch, 2004 ▶). The process of creating the theoretical misorientation table for rotation twins relies on rotational operations and symmetry operations of point groups. In a specific crystal, the transformation matrix ***L*** is obtained from the lattice constants by equation (2). The directions of all symmetry-equivalent twin axes **t** in the crystallographic coordinate system are converted to the corresponding indices **t** ^o^ in the orthonormal coordinate system by equation (3):
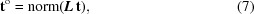
where norm means normalization of a vector. The resulting angle–axis pairs {θ, **t** ^o^} are then converted to a group of rotation matrices ***R*** by combining equations (5) and (6).

For completeness, one must take into account the rotational symmetry operations of the respective point group, which can be expressed as a set of rotation matrices ***S*** (Kocks *et al.*, 2000 ▶). Therefore, the matrices ***X*** describing the full rotational relationships between twin and parent orientations are obtained as
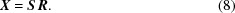
Subsequently, the rotational relationships between twin components are represented as a group of rotation angle–axis pairs {θ, **u** ^o^} derived from matrices ***X*** by inverse operations of equations (6) and (5) (Brannon, 2002 ▶). It is worth noting that the indices **u** ^o^ are in the orthonormal coordinate system. They are then transformed into the corresponding indices **u** in the lattice coordinate system by applying equation (3). Through the procedure executed above, the look-up table of correlation between rotation twin pairs is generated, involving a group of rotation angles and corresponding rotation axes {θ, **u** ^o^}.

### 2.3. Calculating look-up tables for reflection twins   

A reflection twin is defined as two twin components brought into coincidence by a mirror operation along a well defined plane. Previous efforts to derive the correspondence matrices related to the mirror twin–parent lattice orientation (Calbick & Marcus, 1967 ▶; Bevis & Crocker, 1968 ▶; Niewczas, 2010 ▶) were restricted to cubic and hexagonal systems and did not include the intrinsic rotational symmetry of their point groups. Here we show an alternative way to implement a look-up table, which is valid for all seven crystal systems and takes rotational symmetry into account.

With respect to a specific reflection twin, the indices {*p*_1_ *p*_2_ *p*_3_} denoting the family of the twin planes in crystallographic coordinates are converted to the corresponding indices {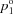 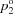 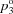} in orthonormal space utilizing the transformation matrix ***L*** by equation (4). In linear algebra, the Householder transformation is introduced to describe a reflection (Householder, 1958 ▶):
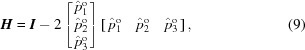
where ***H*** is the Householder matrix, ***I*** is the identity matrix, and the unit vector [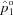 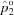 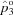] is obtained by normalizing the indices of each plane in {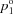 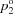 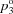}. The determinant of the Householder matrix ***H*** is −1, indicating that a mirror operation changes the chirality of the coordinate system. Since as stated above Laue diffraction is insensitive to chirality, it is safe to multiply ***H*** by −1 without introducing a change in the orientation of the crystal. It can be shown that det(−***H***) = 1 (det means the matrix determinant) and [−***H***]^−1^ = [−***H***]^T^, which identifies −***H*** as a rotation matrix.

Similar to the case of rotation twins, the rotational symmetries of the point group need to be taken into account. Thus the correspondence matrices ***X*** linking twin and parent lattices can be expressed as
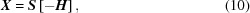
where ***S*** is, again, the group of rotation matrices expressing the rotational symmetry of the point group in a given crystal. Finally, each rotation matrix in ***X*** is transformed into a pair of angle–axis {θ, **u**} in the lattice coordinate system following the same route as for the rotation twin.

### 2.4. Experimental identification of twinning   

As outlined in the previous section, a look-up table for orientation relationships between two twin components can be established as long as all the twin elements (twin axis or twin plane) in a specific crystal are known. The existing twin mode thereafter is identified utilizing a ‘look-up’ table method by comparing the misorientation measurements from µXRD scans with the theoretical rotation angle–axis pairs in the look-up table.

#### 2.4.1. Misorientation measurements from XMAS   

The misorientation matrix Δ***G*** between any two pixels in a µXRD scan from the *XMAS* outputs [***G***_1_ and ***G***_2_, as explained in equation (1)] is deduced as follows:
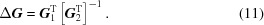
From the inverse operations of equations (5) and (6), the misorientation matrix Δ***G*** is represented as a rotation angle–axis pair {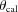, [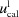 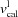 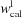]} (the subscript cal means the calculation results, and the superscript l denotes the laboratory coordinate system), and the rotation axis can be transformed into [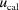 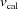 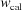] in the lattice coordinate system through a similar transformation operation with equation (3):
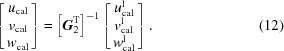
The misorientation of any two adjacent grains is expressed as a rotation angle–axis pair from a µXRD measurement. By comparing measured values with the calculated angle–axis pairs in the look-up table for each measured position pair, twins are unambiguously identified within a specified tolerance range.

#### 2.4.2. Tolerance for determination of twinning types   

The look-up table, involving a set of rotation angle–axis pairs, reveals the rotational relationship between two twin components. On the other hand, two adjacent grains are characterized as a pair of twins as long as the following two conditions are satisfied simultaneously: first, the misorientation angle θ_cal_ between the two grains must be equal to one of the theoretical rotation angles defined in the look-up table; second, the rotation axis **u**_cal_ of two adjacent orientations must be parallel to the theoretical axis corresponding to the rotation angle.

Before applying this method to real samples, it is necessary to consider the potential deviations of the calculated θ_cal_ and **u**_cal_ values from the theoretical case. One contribution arises from the orientation resolution of the experimental technique (∼0.01° for µXRD) and calculation errors. Secondly, a material subjected to a mechanical/electrical/magnetic/temperature field may exhibit small changes of the lattice parameters and/or grain rotation from the strain-free state. For example, deformation twinning is known to be accompanied by grain rotations arising from the accumulation of geometrically necessary dislocations (GNDs) (Straumanis, 1949 ▶). As a result, the X-ray diffraction peaks from such materials will be either broadened or split. Consequently, the measurement accuracy of the crystal orientation is reduced.

Because of inherent uncertainties and errors in the crystal orientation measurements, it is normal that between a pair of twinned orientations the experimentally observed misorientation is slightly different from the theoretical rotation angle, and the rotation axis is not exactly parallel to the theoretical direction. To account for these possibilities in terms of the algorithm discussed, two orientations are defined as a twin pair when
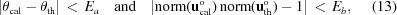
where the subscript th denotes the theoretical values, 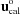 and 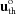 are the corresponding axes in orthonormal coordinates converted from 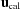 and 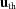 by equation (3), and *E_a_* and *E_b_* are open input variables defining the deviation angles between the measured misorientation angle–axis pairs and the theoretical values, depending on the resolution limit of the technique, sample preparation history, defect density *etc*.

## 3. Applications   

Scanning Laue µXRD experiments were conducted on beamline 12.3.2 at the Advanced Light Source (ALS) of Lawrence Berkeley National Laboratory (LBL). The samples, mounted on a precision translation stage, were scanned at 45° relative to the incident beam with an energy bandpass between 5 and 24 keV at the focal spot (about 1 × 1 µm). At each scanning position, a Laue diffraction pattern was recorded with a PILATUS 1M detector located above the sample in reflection geometry at 90° with respect to the incident X-ray beam. More details of the µXRD experiment setup can be found elsewhere (Kunz *et al.*, 2009 ▶). The obtained Laue patterns were indexed using the software package *XMAS* (Tamura *et al.*, 2003 ▶) after calibrating the diffraction geometry. The procedure provides an accurate measure of spatially resolved grain orientations. By comparing the orientation measurements with the look-up table for a certain twin mode pixel by pixel, one can determine the types of crystal twinning unequivocally and therefore the spatial distribution of twin boundaries. Here we give several examples of the applications of the look-up table described above.

### 3.1. Identification of the Dauphiné twin in quartz – a general case of rotation twinning   

The occurrence of the Dauphiné twin in trigonal α-quartz (space group *P*3_1_21/*P*3_2_21) is often related to the α–β phase transformation (Van Tendeloo *et al.*, 1975 ▶) or deformation processes (Zinserling & Schubnikow, 1933 ▶). The Dauphiné twin law is a rotation twin related to a 180° rotation around the 〈0001〉 axes. Given the space-group symmetry, the twin axes actually consist of two equivalent axes [0001] and [000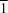]. However, for simplicity, the former is used to explain the algorithm. To formulate the look-up table for the Dauphiné twin, we first derive the transformation matrix ***L*** from equation (2) using the lattice parameters of *a* = 4.921 Å and *c* = 5.416 Å (Glinnemann *et al.*, 1992 ▶):
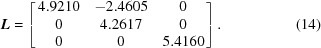
By equation (7), the normalized indices of the twin axis in the orthonormal coordinate system are also [001]; then the angle–axis pair {180°, [001]} is converted to a rotation matrix ***R*** by applying equations (5) and (6):
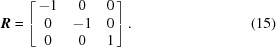



To describe the twinning correlations comprehensively, one must multiply the rotation matrix ***R*** by each of the six rotation matrices ***S*** belonging to point group 32 (Kocks *et al.*, 2000 ▶), and thus six rotational matrices ***X*** are obtained and represented as six angle–axis pairs {θ, **u** ^o^} (Table 2 ▶). Finally, these axes are transformed into the lattice coordinate system by applying equation (3) and denoted as Bravais–Miller indices.

The quartz sample demonstrated here was from the San Andreas Fault Observatory at Depth (Chen *et al.*, 2015 ▶). A thin section was prepared by mounting a slab with epoxy on a glass slide, with subsequent mechanical grinding and polishing to ∼30 µm thickness. A 320 × 240 µm area was scanned with the µXRD technique with a step size of 4 µm and then analyzed using *XMAS*. Owing to the uncertainty in unequivocally indexing a Laue pattern of trigonal crystals, *XMAS* may give a wrong indexing with an orientation ambiguity of 60 or 180° around the *c* axis. Therefore, the output data from *XMAS* were rechecked using an improved peak-intensity-fitting method (Chen *et al.*, 2012 ▶).

**Figure 2:**
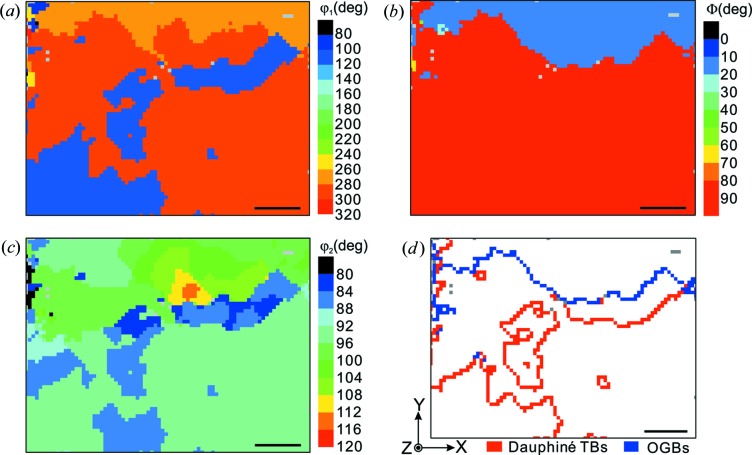
The crystallographic orientation maps of the α-quartz sample indicated by Euler angles (*a*) ϕ_1_, (*b*) Φ and (*c*) ϕ_2_ in the Bunge setting. (*d*) The distribution of Dauphiné twin boundaries denoted in red and ordinary grain boundaries in blue. Scale bars represent 50 µm.

The orientation of the scanned area is represented in Figs. 2 ▶(*a*)−2 ▶(*c*) by Euler angles ϕ_1_, Φ and ϕ_2_ in Bunge notation (Bunge & Morris, 1982 ▶), respectively. The grey pixels indicate the failure of orientation indexing from *XMAS*, either because these positions correspond to a different mineral phase with too few reflections or because the lattice at these spots contains too many defects. A pixel-to-pixel misorientation examination and comparison with the last column of Table 2 ▶ suggest that both OGBs and Dauphiné TBs are detected in the scanned area, and their distribution is shown in Fig. 2 ▶(*d*) with blue and red curves, respectively. In this example, a 2° tolerance for both *E_a_* and *E_b_* was employed. It is worth noting that the misorientation between the parent and twin domains here is 180° around the [0
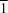
10] axis. In other words, one would probably miss those Dauphiné twin boundaries if not paying attention to the rotational symmetry matrices ***S*** when deducing the look-up table.

### 3.2. Identification of spinel twin in cubic crystals – a general case of reflection twinning   

Table 3 ▶ summaries the theoretical misorientation angles and corresponding axes for the {111}〈112〉 twin in face-centered cubic (f.c.c.) crystals (spinel twin law). In this case, the angle–axis pairs {60°, 〈111〉}, {180°, 〈111〉}, {180°, 〈112〉} and {70.53°, 〈011〉} have been reported in the previous literature (Santamarta & Schryvers, 2004 ▶; Zhu *et al.*, 2005 ▶), while {109.47°, 〈011〉}, {131.81°, 〈012〉} and {146.44°, 〈113〉}, which are seldom referred to by previous studies, are also discovered by taking the rotational symmetry into account.

**Figure 3:**
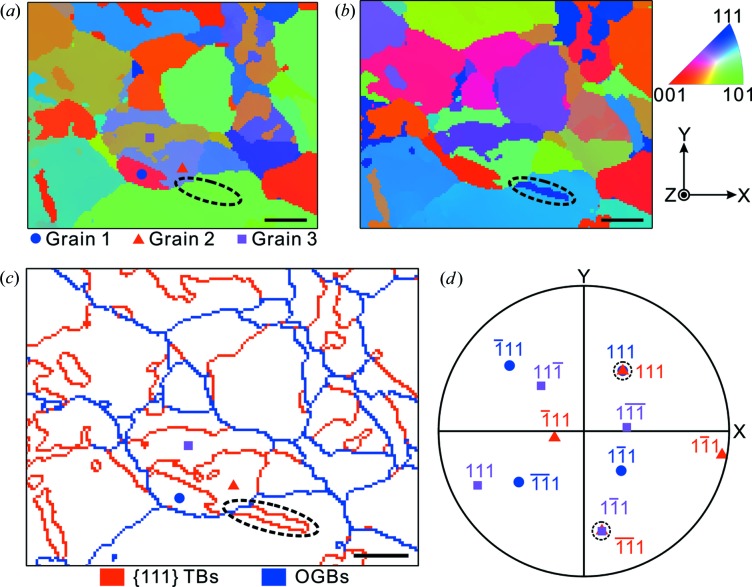
The inverse pole figure maps of the SS304 sample along (*a*) the normal of the sample surface and (*b*) the *y* axis, with the corresponding color code shown at the top right corner. (*c*) The distribution of {111} TBs delineated in red and OGBs in blue. (*d*) {111} stereographic projections of the three selected grains marked in (*a*) and (*c*), and the overlapped poles denoted by dashed circles. Scale bars represent 20 µm.

An area of 150 × 119 µm was scanned with 1 µm step size on a piece of annealed and polished stainless steel 304 (SS304) with the micro-focused X-ray beam (Lupinacci *et al.*, 2015 ▶). After indexing all 17 850 Laue patterns with *XMAS* automatically, the inverse pole figure (IPF) maps were plotted. The crystallographic orientations in the direction normal to the sample surface and parallel to the *y* axis are shown in Figs. 3 ▶(*a*) and 3 ▶(*b*), respectively. Comparing the experimental measure­ments and theoretical misorientation angle–axis pairs, the distribution of various boundary types is drawn in Fig. 3 ▶(*c*), where {111} TBs are delineated in red and the OGBs in blue. The tolerances *E_a_* and *E_b_* were both set at 1° in this example. In addition, it is worth noting that the grain information from only one IPF map may be misleading. For example, the twin boundary circled in Fig. 3 ▶(*c*) is almost invisible in the IPF map along the sample surface normal in Fig. 3 ▶(*a*), while it is clearly seen in Fig. 3 ▶(*b*).

In order to double check the output of the automated twinning identification approach, the {111} stereographic projection of the three adjacent grains marked in Figs. 3 ▶(*a*) and 3 ▶(*c*) is plotted in Fig. 3 ▶(*d*). The (111) poles of Grain 1 and Grain 2 overlap, so that they follow the {60°, [111]} rotation angle–axis pair, while the (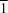 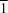
1) pole of Grain 2 is coincident with the (1
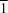
1) pole of Grain 3, corresponding to the {131.81°, [02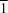]} rotation angle–axis pair listed in Table 3 ▶. As mentioned above, the latter case here is easily overlooked if not considering the symmetry matrices ***S***.

### 3.3. Multiple twinning modes in hexagonal metals   

**Figure 4:**
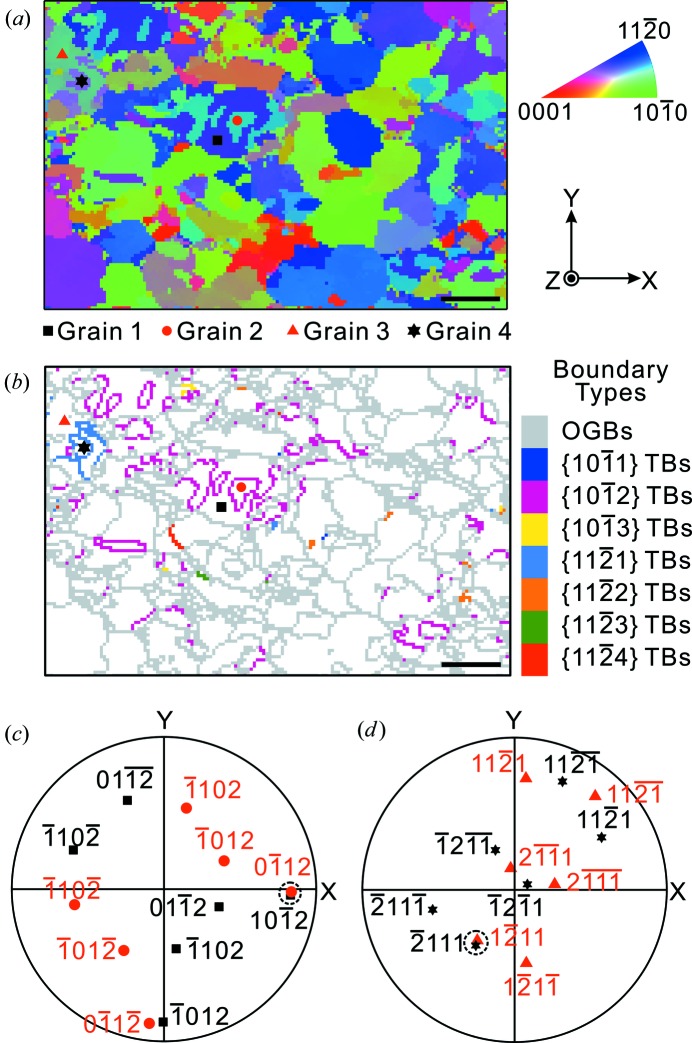
(*a*) The inverse pole figure map along the surface normal of the AZ31 sample. (*b*) The spatial distribution of seven different types of twin boundaries and ordinary grain boundaries. Stereographic projections of (*c*) {10
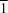
2} poles of Grain 1 and Grain 2, and (*d*) {11
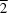
1} poles of Grain 3 and Grain 4, as marked in (*a*) and (*b*). Dashed circles in (*c*) and (*d*) refer to the overlapped poles. Scale bars represent 50 µm.

Seven twinning modes have been reported in hexagonal close-packed metals, namely {10
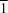
1}〈10
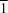
2〉, {10
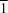
2}〈10
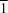
1〉, {10
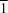
3}〈30
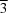
2〉, {11
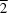
1}〈11
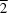
6〉, {11
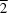
2}〈11
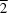
3〉, {11
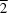
3}〈11
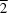
2〉 and {11
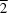
4}〈22
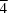
3〉. In order to test the resolution/capability of our automated approach, a magnesium (Mg) AZ31 alloy was rolled to about 3% reduction and then cut and polished for µXRD study. Fig. 4 ▶(*a*) shows the IPF map of a 450 × 300 µm area along the sample surface normal. The scanning step size here was 3 µm. From a comparison of misorientation between each two adjacent scanning spots with the theoretical conditions listed in Table 4 ▶, we obtain the spatial distribution of different types of TBs and OGBs plotted in Fig. 4 ▶(*b*) using the tolerance of 3° both for *E_a_* and for *E_b_*. As illustrated in Fig. 4 ▶(*b*), one of the major benefits of this automated twinning identification algorithm is the immediate representation of the specific twin or even multiple twin types and corresponding spatial distribution by handling a tremendous number of data points in µXRD maps.

To scrutinize the boundaries in Fig. 4 ▶(*b*), one should find some discrete pixels indicating a certain twin type along an ordinary grain boundary, because the Laue diffraction peaks from the highly plastically deformed region become fuzzy and streaked, so that the crystal orientation is not measured accurately.

The output results are rechecked by two means: one through the stereographic projection approach, the other by comparing with the previously reported matrix method (Cheneau-Späth *et al.*, 1994 ▶). The four grains marked in Figs. 4 ▶(*a*) and 4 ▶(*b*) are selected as examples. From the stereographic projections in Figs. 4 ▶(*c*) and 4 ▶(*d*), it is seen that one of the {10
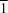
2} poles of Grain 1 overlaps with one of Grain 2, while one of the {11
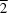
1} poles of Grain 3 appears at the same position for Grain 4, leading to the conclusion that Grain 1 and Grain 2 are a pair of {10
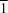
2} twins and Grain 3 and Grain 4 are twinned along the {11
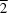
1} plane. The orientation matrices of these four grains are expressed as
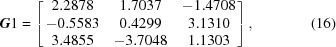


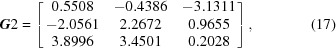


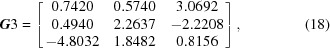


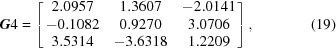
respectively. For {10
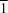
2} and {11
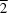
1} twins, the transformation matrices derived in previous literature (Cheneau-Späth *et al.*, 1994 ▶) are
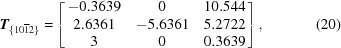


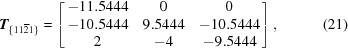
respectively. Imposing them onto ***G***1 and ***G***3, the calculated ***G***2 and ***G***4 are
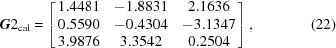


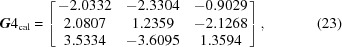
respectively. It is obvious that the calculated orientation matrices can also be written as
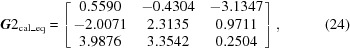


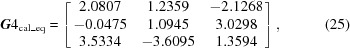
owing to the sixfold rotational symmetry in hexagonal crystals. Upon comparison, taking into account experimental uncertainties, the orientation matrices ***G***2_cal_eq_ and ***G***4_cal_eq_ are in good agreement with the measured results. More grains were selected for a similar manual check, which confirmed that the output results from the automated approach are reliable.

### 3.4. Identification of reflection twins in highly deformed calcite   

**Figure 5:**
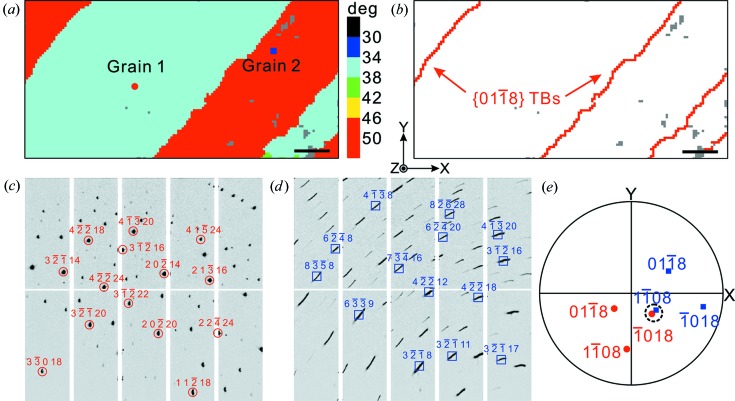
(*a*) Crystallographic orientation map of the calcite sample showing the angles between the crystal *c* axis and the surface normal. (*b*) The spatial distribution of {01
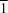
8} twin boundaries. (*c*), (*d*) Two Laue diffraction patterns of the two scanning spots marked in (*a*), and (*e*) the corresponding {01
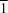
8} stereographic projection. Scale bars represent 30 µm.

The {01
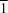
8} mechanical twin, easily visible as lamellar morphologies, is the predominant mechanism of plastic deformation at low temperature in the rhombohedral (space group *R* 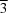
2/*c*) mineral calcite (Ferrill *et al.*, 2004 ▶; Chen *et al.*, 2011 ▶). In our µXRD experiment, a naturally deformed calcite sample was scanned at a 2 µm step size over an area of 280 × 140 µm. A coarse grain with several parallel twin domains can be seen from the orientation map of the angles between the crystal *c* axis and the sample surface normal shown in Fig. 5 ▶(*a*). Setting the tolerance values *E_a_* and *E_b_* to 5 and 5.5° in our twin identification method, the unambiguous {01
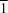
8} reflection twin is determined. The spatial distribution of twin boundaries is delineated in Fig. 5 ▶(*b*). It is worth noting that the tolerances *E_a_* and *E_b_* were set to relatively large values owing to the distortion and rotation of the crystals caused by severe plastic deformation of the sample, which could also be seen from diffraction patterns. As shown in Figs. 5 ▶(*c*) and 5 ▶(*d*), the two Laue patterns corresponding to the scanning spots marked in Fig. 5 ▶(*a*) exhibit very different characteristics. The pattern of Grain 1 is relatively sharp, while Grain 2 shows obvious streaking in Laue peaks caused by the abundant presence of geometrically necessary dislocations (Ice & Barabash, 2007 ▶). In addition, from the {01
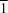
8} stereographic projection shown in Fig. 5 ▶(*e*), the (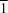
018) pole of Grain 1 and (1
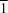
08) pole of Grain 2 are close but not exactly coincident. To summarize, the selected threshold level has a significant impact on characterization of the boundary types. If grain boundaries are misindexed as twins, *E_a_* and *E_b_* will need to be reduced, while if twins are mistaken as grain boundaries, it is necessary to set larger threshold values. In general, to fully account for any suspected ambiguity, it is relatively straightforward to run the deformation twin identification routine multiple times over a small range of *E_a_* and *E_b_* parameters.

## 4. Conclusions   

A fully automated approach for determination of crystal twinning modes is developed. This offers a fast and reliable methodology to identify the exact nature of twinning based on synchrotron Laue µXRD orientation maps. The major advantage highlighted by this work is the new algorithm for generating the theoretical look-up table which contains all the possible rotational correlations for established twin laws. To compare the misorientation angle and axis between adjacent scanning spots with the established look-up table, the unambiguous types of crystal twinning and spatial distribution of twin boundaries are automatically identified through an appropriate selection of threshold values. This ‘look-up’ method is also valid for twinning determination in other raster scanning crystal orientation mapping techniques such as EBSD.

## Figures and Tables

**Table 1:** Crystal systems and Laue groups

Crystal systems	Laue groups
Triclinic	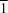
Monoclinic	2/*m*
Orthorhombic	*mmm*
Tetragonal	4/*m*, 4/*mmm*
Trigonal	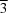 , 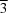 *m*
Hexagonal	6/*m*, 6/*mmm*
Cubic	*m* 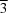 , *m* 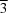 *m*

**Table 2:** The rotational parameters involved in the derivation process of the look-up table for Dauphin twinning (twin element is {180, [0001]}) in -quartz

Symmetry operators ***S*** of point group 32	Rotation matrices ***X*** = ***S*** ***R***	Angleaxis pairs in **O-a** ^o^ **b** ^o^ **c** ^o^ {, **u** ^o^}	Angleaxis pairs in **O-abc** {, **u**}
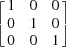	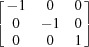	{180, [001]}	{180, [0001]}
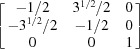	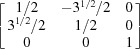	{60, [001]}	{60, [0001]}
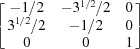	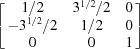	{60, [00 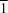 ]}	{60, [000 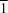 ]}
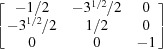	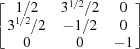	{180, 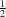 [3^1/2^10]}	{180, [10 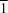 0]}
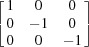	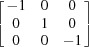	{180, [010]}	{180, [0 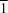 10]}
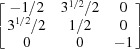	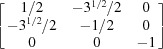	{180, 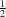 [ 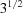 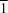 0]}	{180, [ 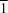 100]}

**Table 3:** The look-up table describing the orientation relationships of spinel twinning in f.c.c. crystals (twin element is {111}112)

Rotation angle	Rotation axis
60	111
70.53 or acos(1/3)	011
109.47 or acos(1/3)	011
131.81 or acos(2/3)	012
146.44 or acos(5/6)	113
180	111, 112

**Table 4:** The look-up table summarizing the twinparent correlations in seven twinning systems in a hexagonal crystal, where the rotation axes are represented as either direction indices *uvtw* or the normal of the plane indices {*hkil*}, = *c*/*a*

		Rotation axis			Rotation axis	
Twinning system	Rotation angle ()	*uvtw*	{*hkil*} normal	Rotation angle ()	*uvtw*	{*hkil*} normal
{10 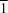 1}10 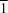 2	180	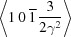	{10 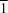 1}	180	10 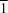 2	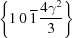
	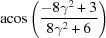	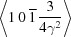	{20 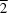 1}	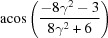	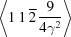	{22 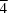 3}
	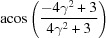	11 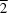 0	{11 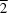 0}	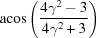	11 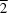 0	{11 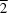 0}
	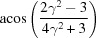	10 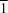 1	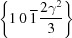	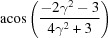	11 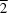 3	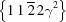

{10 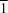 2}10 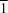 1	180	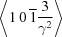	{10 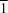 2}	180	10 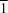 1	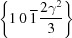
	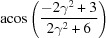	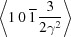	{10 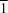 1}	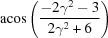	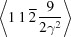	{11 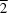 3}
	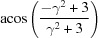	11 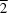 0	{11 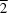 0}	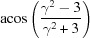	11 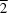 0	{11 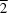 0}
	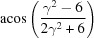	20 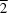 1	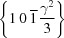	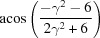	22 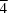 3	{11 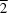 ^2^}

{10 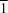 3}30 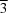 2	180	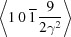	{10 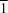 3}	180	30 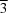 2	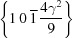
	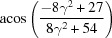	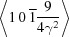	{20 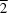 3}	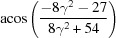	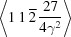	{22 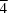 9}
	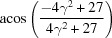	11 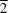 0	{11 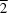 0}	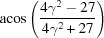	11 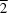 0	{11 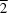 0}
	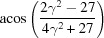	30 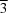 1	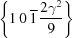	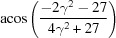	11 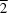 1	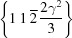

{11 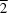 1}11 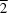 6	180	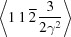	{11 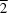 1}	180	11 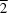 6	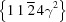
	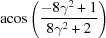	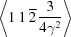	{22 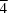 1}	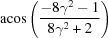	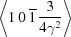	{20 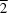 1}
	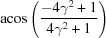	10 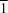 0	{10 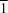 0}	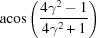	10 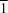 0	{10 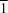 0}
	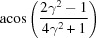	11 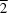 3	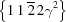	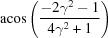	10 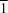 3	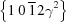

{11 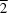 2}11 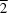 3	180	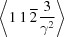	{11 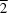 2}	180	11 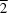 3	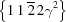
	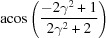	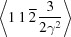	{11 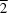 1}	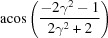	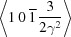	{10 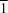 1}
	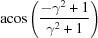	10 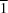 0	{10 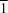 0}	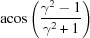	10 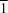 0	{10 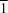 0}
	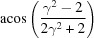	22 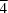 3	{11 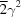 }	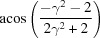	20 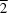 3	{10 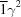 }

{11 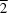 3}11 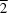 2	180	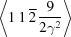	{11 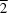 3}	180	11 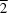 2	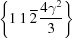
	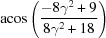	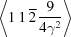	{22 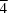 3}	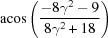	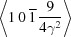	{20 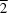 3}
	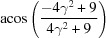	10 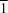 0	{10 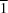 0}	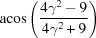	10 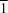 0	{10 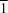 0}
	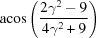	11 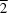 1	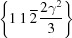	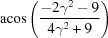	10 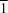 1	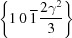

{11 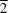 4}22 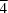 3	180	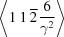	{11 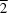 4}	180	22 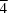 3	{11 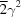 }
	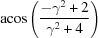	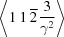	{11 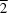 2}	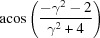	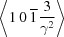	{10 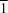 2}
	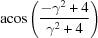	10 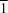 0	{10 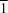 0}	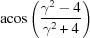	10 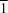 0	{10 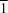 0}
	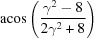	44 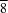 3	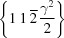	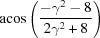	40 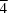 3	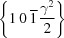
